# Conservation and divergence of bHLH genes in the calcisponge *Sycon ciliatum*

**DOI:** 10.1186/s13227-016-0060-8

**Published:** 2016-10-14

**Authors:** Sofia A. V. Fortunato, Michel Vervoort, Marcin Adamski, Maja Adamska

**Affiliations:** 1 Sars International Centre for Marine Molecular Biology, University of Bergen, Bergen, Norway; 2 Institut Jacques Monod – CNRS, Université Paris Diderot, 75005 Paris Cedex 13, France; 3 ARC Centre for Excellence for Coral Reef Studies, James Cook University, Townsville, QLD 4811 Australia; 4 Research School of Biology, Australian National University, Canberra, Australia

**Keywords:** bHLH, Transcription factors, Developmental regulatory genes, *Sycon ciliatum*, Sponges, Evolution

## Abstract

**Background:**

Basic Helix-Loop-Helix (bHLH) genes encode a large family of eukaryotic transcription factors, categorized into six high-order groups: pan-eukaryotic group B involved in regulation of cell cycle, metabolism, and development; holozoan-specific groups C and F involved in development and maintenance of homeostasis; and metazoan-specific groups A, D and E including well-studied genes, such as *Atonal*, *Twist* and *Hairy*, with diverse developmental roles including control of morphogenesis and specification of neurons. Current scenarios of bHLH evolution in animals are mainly based on the bHLH gene set found in the genome of demosponge *Amphimedon queenslandica.* In this species, the majority of the 21 identified bHLH genes belong to group B, and the single group A gene is orthologous to several neurogenic bilaterian subfamilies, including *atonal* and *neurogenin*.

**Results:**

Given recently discovered differences in developmental toolkit components between siliceous and calcareous sponges, we have carried out genome-wide analysis of bHLH genes in *Sycon ciliatum*, an emerging calcisponge model. We identified 30 bHLH genes in this species, representing 12 individual families, including four group A families not found in *Amphimedon*, and two larger family groupings. Notably, the families represented in *Sycon* are only partially overlapping with those represented in *Amphimedon.* Developmental expression analysis of a subset of the identified genes revealed patterns consistent with deeply conserved roles, such as specification of sensory cells by *Atona*-*related* and stem cells by *Myc* genes.

**Conclusions:**

Our results demonstrate independent gene loss events in demosponges and calcisponges, implying a complex bHLH toolkit in the last common metazoan ancestor.

**Electronic supplementary material:**

The online version of this article (doi:10.1186/s13227-016-0060-8) contains supplementary material, which is available to authorized users.

## Background

Basic Helix-Loop-Helix (bHLH) genes constitute a large family of transcription factors (TFs) that are widely found in eukaryotes [1–4]. The bHLH domain is about 60 amino acid long and consists of a DNA-binding basic region (b) followed by a domain comprising two α-helices separated by a variable loop region (HLH), which is involved in the formation of homodimeric or heterodimeric complexes [1]. Based on structural and biochemical properties, bHLH TFs have been categorized into six high-order groups [5, 6]. Group A, which is specific to metazoans [4, 7], mainly contains bHLH TFs, usually with no other conserved domains, which are involved in various developmental processes, including neurogenesis and myogenesis [1]. In the phylogenetic analyses, many of the class A subfamilies are found in either of two monophyletic groups, the so-called Atonal-related and Twist-related groups [4, 6]. Group B includes, in addition to several metazoan proteins, the vast majority of the non-metazoan bHLH TFs, including all fungi and plant ones [2, 3]. Group B proteins, which often contains leucine zippers in addition to the bHLH domain, display functions in a wide variety of processes, including cell cycle, cell and organismal metabolism, and development [1, 8]. Group C proteins contain a ‘PAS’ (Per-AHR nuclear translocator (ARNT)-Sim) domain that provide an additional dimerization motif and are involved in developmental signalling and environmental homeostasis [9]. Whereas bHLH-PAS proteins were initially thought to be specific to metazoans, a gene encoding a protein from this high-order group has been found in the filasterean *Capsaspora owczarzaki* [7]. The metazoan-specific group D includes HLH protein that lacks the basic domain (hence is unable to bind DNA) and acts as antagonists of group A bHLH proteins [1]. Proteins of the group E, known as Hairy and Enhancer of split-related proteins, are only found in metazoans and mainly act as developmental regulators [10]. Most of these proteins contain an additional domain, named the ‘orange’ domain, as well as a C-terminal WRPW peptide, both involved in protein–protein interactions [10]. Group F corresponds to the COE (Collier/Olf/EBF) proteins that are found in metazoans and in *Capsaspora owczarzaki* [11]. These proteins lack the basic domain and are characterized by the presence of the ‘COE’ domain, involved in both dimerization and DNA binding [12].

The identification of the putative full set of bHLH genes in many different metazoan and closely related non-metozoan genomes [4, 6, 7, 11, 13–17] allowed to better understand how the repertoire of bHLH genes evolved in animals. These data led to the proposal that bHLH genes underwent three main phases of expansion: a first one prior to metazoan diversification, a second one after the divergence between sponges and other metazoans, and a third one after the divergence between cnidarians and bilaterians [4, 18]. This scenario of bHLH evolution crucially depends on the data obtained in the sponge *Amphimedon queenslandica* (Class Demospongiae), as it is based on the animal phylogeny in which sponges constitute the sister group to all other metazoans, and which has been supported by some but not all recent phylogenomic studies [19–23]. *Amphimedon* owns 21 bHLH genes [4, 17], and this is much less than cnidarians and bilaterians [4, 15]. Most *Amphimedon* bHLHs are ortholog to defined bilaterian bHLH subfamilies and belong to the high-order group B [4, 17]. This therefore led to the suggestion that an important part of the diversification of bHLHs in animals occurred after the split between sponges and other animals. This is particularly the case for group A bHLHs as a single gene from this group was found in *Amphimedon* and was shown to be the ortholog of several bilaterian subfamilies, such as the neurogenic genes *atonal*, *neurogenin*, *NeuroD*, and *olig* [24]. Interestingly, although sponges lack neurons, the *Amphimedon* group A bHLH (*AmqbHLH1*) was shown to be expressed in a putative sensory cell type during development. Moreover, functional analysis of *AmqbHLH1* demonstrated its ability to induce formation of ectopic neurons in *Xenopus* and ectopic sensory organs in *Drosophila*, suggesting a conserved involvement of this gene in the development of sensory and neural-like cells [24].

Single species, such as *Amphimedon*, cannot be considered as representative of a whole phylum, in particular a very ancient one such as sponges. Moreover, we cannot ascertain whether the reduced number of bHLHs and the absence of most group A genes in *Amphimedon* represent the ancestral situation or may be due, at least in part, to secondary gene losses. Several recent studies have shown that another sponge, the calcisponge *Sycon ciliatum* (Class *Calcarea,* Subclass *Calcaronea*), displays many more members of several developmental gene families than *Amphimedon* and possesses orthologs of some bilaterian genes not found in the *Amphimedon* genome [25–30]. *Sycon ciliatum* is an emerging model for comparative developmental biology studies [31]. Embryonic and postembryonic development of calcaronean sponges has been well described [29, 32–35]. Embryogenesis is internal and occurs in the space (mesohyl) between the inner (choanoderm) and outer (pinacoderm) epithelial layers of the adult sponge. Cleavage is stereotypic and leads, after cell differentiation and tissue inversion, to the formation of a swimming larva composed of numerous ciliated micromeres, a lower number of macromeres and four cross cells interspersed among micromeres. The larva has a tetra-radial symmetry due to the position of the cross cells. The micromeres and macromeres contribute, after metamorphosis, to the formation of the choanoderm and pinacoderm of the radially symmetrical juvenile sponge, respectively. In contrast, the cross cells, which are thought to be the larval sensory cells [36], disappear upon settlement [32]. This well-understood origin and fate of larval cells provides solid background to analyse gene expression patterns in calcaronean sponges. Intriguingly, expression of several developmental regulatory genes is consistent with homology of sponge and eumetazoan cell types, in particular larval sensory cells with neurons and choanocytes with endomesodermal and stem cells [25–27, 29, 31, 37].

In this article, we report genome-wide analysis of bHLH genes in *Sycon ciliatum*. As in case of transcription factors families studied previously, the bHLH repertoires in *Sycon* and *Amphimedon* are very different, in terms of both gene numbers and families represented. Strikingly, developmental expression patterns of the conserved family members are consistent with the previously postulated cell-type homologies.

## Methods

### bHLH sequences identification

Candidate *Sycon ciliatum* bHLH sequences were identified by blastp searches using bHLH proteins from a sample of bilaterian (Human, *Drosophila*, *Lottia gigantea*, and *Capitella teleta*) and non-bilaterian (*Amphimedon queenslandica* and *Nematostella vectensis*) species against the database of protein sequences predicted from previously described transcriptome [38]. We identified as bHLH proteins the *Sycon* sequences that (1) contain a bHLH domain as defined by using NCBI CD-search [39] and (2) allow to retrieve a bHLH protein as best blast hit when used as query in blastp searches against the Human and *Drosophila* Refseq protein databases. The sequences of all the *Sycon* bLH proteins are available in Additional file 1. The same approach was used to identify the bHLH proteins encoded by the genomes of the ctenophores *Pleurobrachia bachei* and *Mnemiopsis leidyi.*

### Phylogenetic analyses

The bHLH domains of a large set of bHLH proteins from various species (see Results for details) were retrieved from the relevant publications [4, 13, 15] and aligned with the bHLH domains of the *Sycon* proteins, using MUSCLE 3.8 [40]. The obtained multiple alignment is available in Additional file 2. Maximum likelihood (ML) analyses were performed with PhyML [41] using the PhyML web server [42] hosted at the Montpellier bioinformatics platform (http://www.atgc-montpellier.fr/phyml/). PhyML analyses were performed using the Le and Gascuel (LG) amino acid substitution model [43], using two rate categories (one constant and four γ rates). Statistical supports for the different internal branches were determined by approximate Likelihood-ratio test (aLRT) and a Bayesian-like transformation of aLRT (aBayes) [44, 45].

### Quantitative expression analysis

Quantitative analysis of the gene expression levels was performed on protein coding sequences identified in *Sycon ciliatum* transcriptome as previously described in [26, 27, 29, 38]. Briefly, gene expression levels were calculated from the RNASeq datasets (ArrayExpress ids E-MTAB-2430, E-MTAB-2431and E-MTAB-2890) using RSEM [46] and DESeq packages [47]. Gene expression levels were calculated as sums of the posterior probability of each read coming from a given gene over all reads, the ‘expected_count’ metrics from RSEM; scaled (normalized) with the size factors of the RNASeq datasets calculated by DESeq. A matrix of the expected_counts for all the coding sequences the datasets is available in Additional file 3. Detection of the differentially expressed genes was performed using the DESeq package (using the negative binomial distribution). Statistical significance of the detection was inferred for genes for which the *p* value adjusted for multiple testing was less or equal 0.1 (the *p* value was adjusted with the Benjamini–Hochberg procedure, the ‘padj’ value from nbinomTest() function from DESeq).

### Samples

*Sycon ciliatum* specimens containing embryos were collected in fjords near Bergen (Norway) and fixed for in situ hybridization as described previously [28].

### Gene amplification and in situ hybridization

Complementary DNA was produced using SuperScript Reverse Transcriptase III (Sigma) and pooled total RNA isolated from tissue samples containing a broad range of developmental stages. cDNA was used as template for PCR reactions. Gene-specific primers were designed for the amplification of 800 bp to 1 kb fragment of *Sycon* bHlH genes for riboprobe synthesis. Probe synthesis and in situ hybridization were performed as described in Fortunato et al. [28].

## Results and discussion

### Identification and phylogenetic analysis of the Sycon bHLH genes

We identified 30 bHLH genes in the fully sequenced genome of *Sycon ciliatum* (Table 1). We performed phylogenetic analyses to assess whether these *Sycon* sequences can be assigned to the previously described subfamilies of bHLH genes [4, 14, 15]. Phylogenetic analyses were conducted using a multiple alignment including the bHLH domains from *Sycon ciliatum* and *Amphimedon queenslandica* (sponges), *Trichoplax adhaerens* (placozoans), *Nematostella vectensis*, *Hydra magnipapillata* and *Acropora digitifera* (cnidarians), *Drosophila melanogaster* and *Daphnia pulex* (bilaterians, arthropods), *Lottia gigantea* (bilaterians, molluscs), *Capitella teleta* (bilaterians, annelids), and *Homo sapiens* (bilaterians, chordates).

**Table 1:** List of all the identified *Sycon* bHLH genes

*Sycon* bHLH	Family	Superfamily	High-order group	Domain(s) additional to bHLH	Linkage
*SciAtonal*-*related*	–	Atonal-related	A	–	
*SciNSCLa*	NSCL	Twist-related	A	–	scaffold 20
*SciNSCLb*	NSCL	Twist-related	A	–	scaffold 20
*SciNSCLc*	NSCL	Twist-related	A	–	scaffold 20
*SciNSCLd*	NSCL	Twist-related	A	–	
*SciHand*	Hand	Twist-related	A	–	
*SciMyoRb*	MyoRb	Twist-related	A	–	
*SciSCLa*	SCL	Twist-related	A	–	scaffold 316
*SciSCLb*	SCL	Twist-related	A	–	
*SciSCL*-*like a*	–	Twist-related	A	–	
*SciSCL*-*like b*	–	Twist-related	A	–	
*SciSCL*-*like c*	–	Twist-related	A	–	
*SciSCL*-*like d*	–	Twist-related	A	–	
*SciSCL*-*like e*	–	Twist-related	A	–	scaffold 310
*SciSCL*-*like f*	–	Twist-related	A	–	scaffold 310
*SciOrphan 1*	–	Twist-related	A	–	scaffold 316
*SciE12/E47a*	E12/E47	–	A	–	
*SciE12/E47b*	E12/E47	–	A	–	
*SciUSF*	USF	–	B	–	
*SciMITFa*	MITF	–	B	–	
*SciMITFb*	MITF	–	B	–	
*SciSREBP*	SREBP	–	B	–	
*SciTF4*	TF4	–	B	–	
*SciMlx*	MLX	–	B	–	
*SciMyc*	MYC	–	B	MYC-N	
*SciARNT*-*like*	–	ARNT + BMAL	C	PAS	
*SciHey*	HEY	–	E	Hairy_Orange	
*SciOrphan2*	–	–	–	–	
*SciOrphan3*	–	–	–	–	
*SciOrphan4*	–	–	–	–	

Expression of genes indicated by bold font was studied by in situ hybridization. Three instances of genomic linkage were observed, and the scaffolds in which these genes are included are indicated

**Fig. 1:**
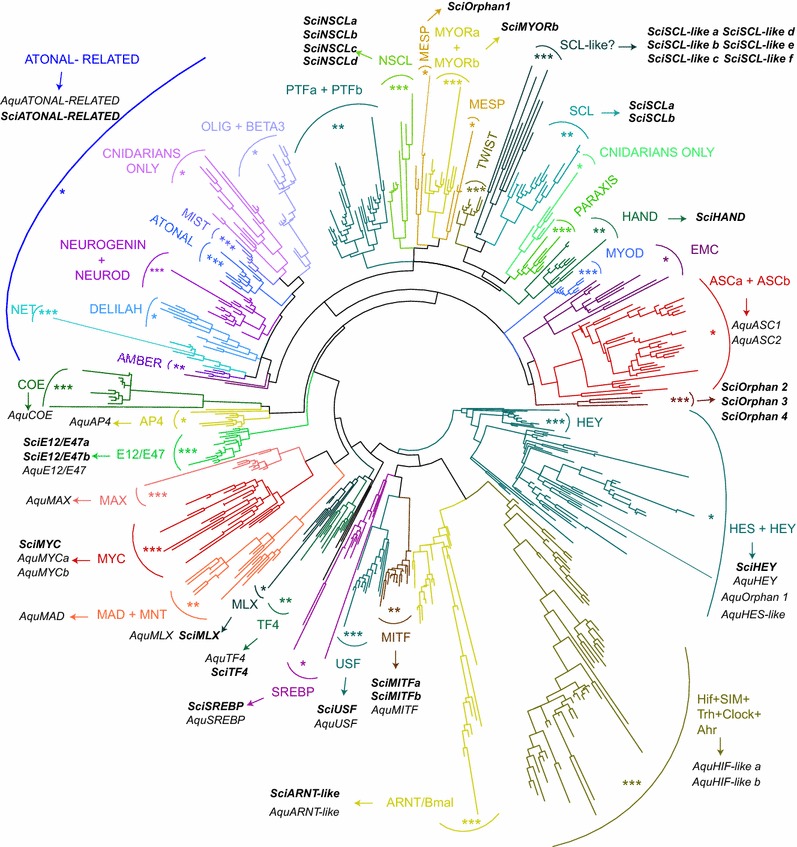
Phylogenetic analysis of the *Sycon* bHLH proteins. An unrooted maximum likelihood (ML) tree is shown. This tree has been constructed using the bHLH domains from *Sycon ciliatum* and *Amphimedon queenslandica* (sponges), *Trichoplax adhaerens* (placozoans), *Nematostella vectensis*, *Hydra magnipapillata* and *Acropora digitifera* (cnidarians), *Drosophila melanogaster* and *Daphnia pulex* (bilaterians, arthropods), *Lottia gigantea* (bilaterians, molluscs), *Capitella teleta* (bilaterians, annelids), and *Homo sapiens* (bilaterians, chordates). Several monophyletic groups are found and are named according to previous studies [4, 14, 15]. The Atonal-related group [4] is also indicated. We listed in the figure the proteins from *Sycon ciliatum* (*Sci*) and *Amphimedon queenslandica* (*Aqu*). The robustness of the nodes that define the different monophyletic groups was assessed by evaluating their statistical supports in the ML analysis. This is represented on the tree by the presence close to the name of the group of *** (aLRT and aBayes values > 0.95), ** (0.95 > aLRT and aBayes values > 0.8), or * (aLRT and aBayes values < 0.8)

We observed all the previously defined bHLH subfamilies and found that 18 of the *Sycon* sequences belong to single defined bHLH subfamilies (12 different families; Fig. 1). These genes were named according to the family they belong to (Table 1). Two *Sycon* bHLHs were found to be associated with two or more subfamilies: one to all Atonal-related subfamilies (this gene was therefore named ‘Atonal-related’) and the other to the BMAL and ARNT subfamilies (this gene was named ‘ARNT-like’; Fig. 1; Table 1; see below for further discussions). Six *Sycon* bHLHs form a monophyletic group that is, within the Twist-related group, associated with the SCL subfamily (Fig. 1)—we named these genes ‘SCL-like’ (Table 1). Finally, we classified four *Sycon* bHLHs as ‘orphans’, because these sequences were not included in any bHLH subfamilies and did not show any consistent association with particular bHLH subfamilies (Fig. 1; Table 1). One of these orphan genes (*Scil*-*Orphan1*), however, clearly belongs to the Twist-related group. Additional file 4: Figure S1, Additional file 5: Figure S2, Additional file 6: Figure S3, Additional file 7: Figure S4, Additional file 8: Figure S5, Additional file 9: Figure S6, Additional file 10: Figure S7, Additional file 11: Figure S8 display phylogenetic trees of the different subfamilies that contain *Sycon* sequences. We have to mention that, due to the short size and high conservation of the bHLH domain, similarly to what has been found in previous studies [4, 14, 15], the statistical supports for some of the monophyletic groups in the phygenetic tree are quite low. This is the case for some subfamilies that include *Sycon* proteins, such as SREBP and MLX (Fig. 1), and we can therefore not rule out that we may have misassigned some of these proteins.

We next looked to the presence of conserved domains (other than the bHLH domain) encoded by the identified *Sycon* genes. Such domains were found in only three cases, and the nature of these domains fully supports the assignation of the corresponding genes, in particular bHLH subfamily (Table 1). Thus, the single *Sycon Myc* gene encodes a Myc-N domain characteristic of this subfamily, the ARNT-like gene a PAS domain only found in group C bHLH proteins, and the *Hey* gene an Orange domain characteristic of Hey and HES/Hairy-related genes. We also examined the genomic localization of the *Sycon* bHLH genes and found three instances of genomic linkage (Table 1). In two cases, the linked genes belong to the same bHLH subfamily and show close relationships in the phylogenetic tree, suggesting that they have been produced by quite recent tandem gene duplications.

Several lines of conclusions can be drawn from these data, in particular when compared with the data on bHLH genes from *Amphimedon* [4, 17]. First, our data allow to increase the number of bHLH genes that were likely present in the last common ancestor (LCA) of sponges, cnidarians, and bilaterians. We indeed found four families (MYORb, SCL, NCL, and HAND; these families have intermediate or high statistical supports in the phylogenetic tree, Fig. 1) that have *Sycon*, but no *Amphimedon* members (Table 2), suggesting that these four types of bHLHs were already present in the LCA of sponges, cnidarians, and bilaterians and have been lost in the lineage leading to *Amphimedon*. If we add these four families to those that have *Amphimedon* (and often *Sycon*) members, we end up with a minimum of 21–22 bHLH types present in the LCA of sponges, cnidarians, and bilaterians (Table 2). This corresponds roughly to one half of the families found in bilaterians (44) [4]. We also identified the bHLH genes present in the genome of two ctenophores, *Pleurobrachia bachei* and *Mnemiopsis leidyi* (12 and 21, respectively). We were, however, unable to assign most of the ctenophore proteins to defined bHLH families, and the inclusion of these sequences in the phylogenetic analysis strongly modified the topology of the phylogenetic tree, disrupting several monophyletic groups including some only composed of bilaterian sequences (not shown). Our analysis of the ctenophore bHLH proteins does therefore not provide additional insights into the evolution of bHLH proteins in animals.

**Table 2:** Number of *Sycon* and *Amphimedon* members of the different bHLH families

Family name	High-order group	*Sycon ciliatum*	*Amphimedon queenslandica*
Achaete-Scute a	A	0	2?
Achaete-Scute b	A		
MyoD	A	0	0
E12/E47	A	2	1
Neurogenin	A	1	1
NeuroD	A		
Atonal	A		
Mist	A		
Amber	A		
Beta3	A		
Oligo	A		
Net	A		
Delilah	A		
Mesp	A	0	0
Twist	A	0	0
Paraxis	A	0	0
MyoRa	A	0	0
MyoRb	A	1	0
Hand	A	1	0
PTFa	A	0	0
PTFb	A	0	0
SCL	A	2	0
NSCL	A	4	0
Myc	B	1	2
Mad	B	0	1
Mnt	B	0	0
Max	B	0	1
USF	B	1	1
MITF	B	2	1
SREBP	B	1	1
AP4	B	0	1
MLX	B	1	1
TF4	B	1	1
Clock	C	0	0
ARNT	C	1	1
Bmal	C		
AHR	C	0	1
Sim	C	0	1
Trh	C		
HIF	C		
SRC	C	0	0
Emc	D	0	0
Hey	E	1	1
Hairy/E(spl)	E	0	1
Coe	F	0	1
Orphans	–	10^a^	1–3
Total number		30	21

^a^Among which 7 belong to the Twist-related superfamily

Second, 18 *Sycon* bHLHs belong to the high-order group A, as compared to two to four in *Amphimedon* (Table 2). Most group A *Sycon* bHLHs belong to the Twist-related group, and four of the constituting subfamilies (MyoRb, Hand, SCL, and NSCL) clearly contain *Sycon* members (Table 2; Additional file 5: Figure S2). This is in sharp contrast with *Amphimedon* in which no such genes were found [4, 17]. This therefore suggests that the Twist-related group, which mostly contains bilaterian tissue-specific developmental regulators, evolved much earlier (before the split between sponges, cnidarians and bilaterians) than previously thought. In contrast, like in *Amphimedon*, we found a single *Sycon* gene belonging to the Atonal-related group (Table 2). Both sponge genes behave as outgroup to all the Atonal-related subfamilies in the phylogenetic tree (Additional file 4: Figure S1), reinforcing the previously proposed hypothesis that all the Atonal-related subfamilies emerged through gene duplications from a single ancestral gene in the cnidarian/bilaterian lineage after its split from sponges [4, 24]. Finally, our data suggest the occurrence of several independent gene losses and duplications in *Sycon* and *Amphimedon*, which is consistent with previous reports [26–30] (reviewed by [25]). There could have been at least six and four gene losses in *Sycon* and *Amphimedon*, respectively. This could, however, be an overestimation because some of these losses concern bHLH subfamilies that have poor statistical supports in the phylogenetic tree (such as ASC and HES) and therefore the inclusion of *Sycon* or *Amphimedon* genes in these subfamilies is questionable. In addition, at least six duplications occurred in the *Sycon* lineage and two in the *Amphimedon* lineage.

We therefore conclude that *Sycon* has a rich complement of bHLH genes, significantly different from that of *Amphimedon*, and which includes several genes whose orthologs in bilaterians are developmental regulators, often with tissue-specific expressions during development.

### Developmental expression of the Sycon bHLH genes

**Fig. 2:**
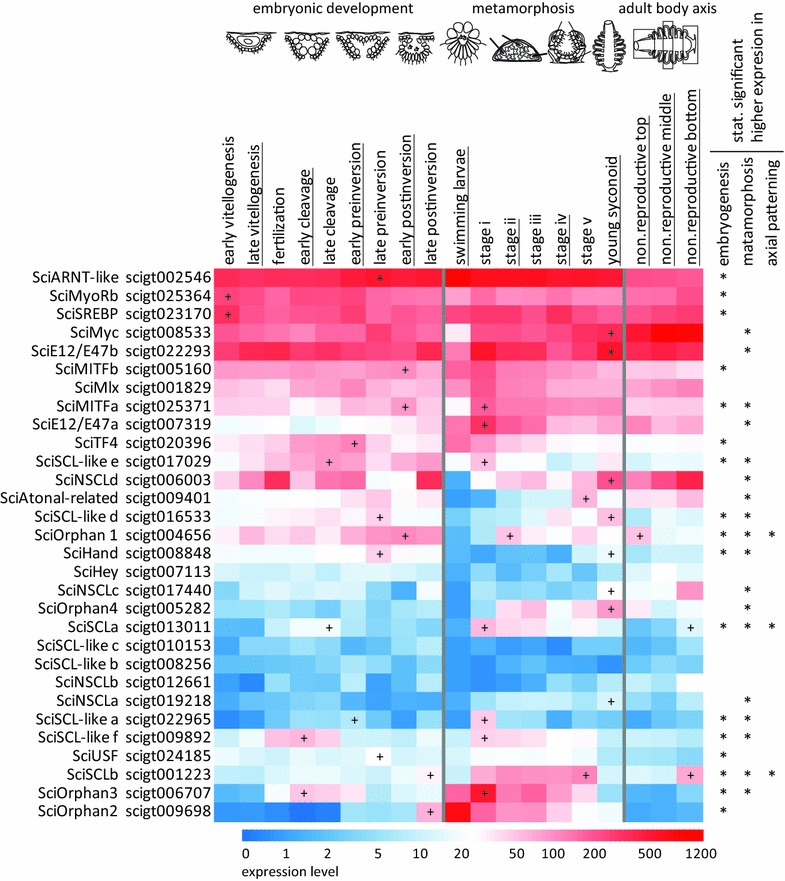
Heatmap representation of expression profiles of *Sycon ciliatum* bHLH genes identified in this study. The expression levels were calculated as described in the methods quantitative analysis section (expected_count from RSEM package normalized between datasets with DESeq package) and then log 10 transformed. The colour scale is from blue (lowest) through white (medium) to red (highest). In each section of the heatmap – ‘embryonic development’, ‘metamorphosis’, and ‘adult body axis’ – the stage at which the gene has the highest expression level and at the same time the expression level is statistically significantly (padj ≤ 0.1) higher than at the reference stage was marked with the ‘+’ symbol. The reference stage for ‘embryonic development’ and ‘adult body axis’ is the ‘non.reproductive middle’, for the ‘metamorphosis’ it is the ‘swimming larvae’. The genes expressed at the statistically significantly higher level in ‘embryonic development’, ‘metamorphosis’, and ‘adult body axis’ than at the reference stage were marked with ‘*’ at the heatmap’s right margin. The stages with underlined names are illustrated. Note that early vitellogenesis to late postinversion samples are mid-body slices, composed of somatic tissues and oocytes or embryos

**Fig. 3:**
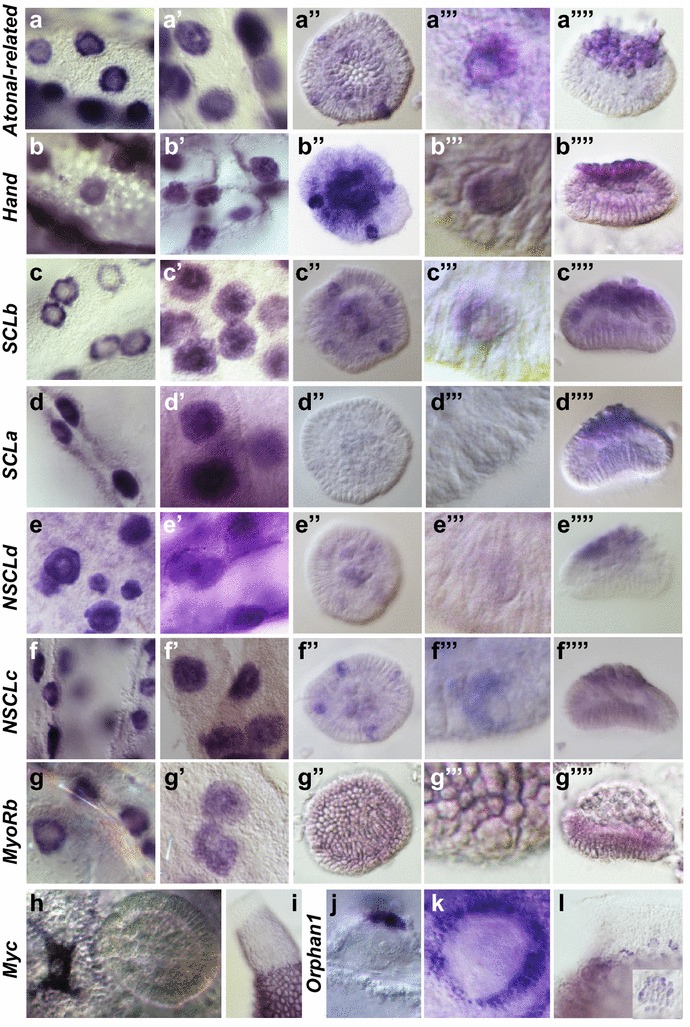
Expression of selected *Syco*n *ciliatum* bHLH genes during embryogenesis. **a–a’’’’**, *Atonal*-*related* expression is detected in the oocytes (**a**), all blastomers during cleavage (**a’**), cross cells in early postinversion stage embryos (**a’’**–**a’’’**), strongly in the macromeres and weakly in the cross cells of late postinversion stage embryos (**a’’’’**); **b–b’’’’**, *Hand* expression is detected in choanocytes and oocytes (**b**), all blastomeres during cleavage (**b’**), strongly in cross cells and macromeres and weaker in micromeres of early postinversion stage embryos (**b’’**–**b’’’**), strongly in macromeres and weakly in other cells of late postinversion stage embryos (**b’’’’**); **c–c’’’’**, *SCLb* expression is detected in oocytes (**c**), strongly in macromeres and weaker in other cell types of cleavage-stage embryos (**c’**), strongly in macromeres and cross cells and weaker in micromeres in postinversion stage embryos (**c’’**–**c’’’’**); **d–d’’’’**, *SCLa* is expressed in oocytes (**d**) and all blastomeres during cleavage (**d’**), not detectable in early postinversion embryos (**d’’**-**d’’’**), and expressed in macromeres in late postinversion stage embryos (**d’’’’**); **e–e’’’**, *NSCLd* expression is expressed in choanocytes, oocytes, and all blastomeres during cleavage (**e**, **e’**), the expression becomes weaker in later embryonic stages, and it is detectable in cross cells (**e’’**, **e’’’**) and macromeres (**e’’**, **e’’’’**) of postinversion stage embryos; **f–f’’’**, *NSCLc* expression is detected in oocytes (**f**), all blastomeres during cleavage (**f’**), in cross cells and macromeres of early postinversion stage embryos (**f’’**, **f’’’**) and in macromeres of late postinversion stage embryos (**f’’’’**); **g–g’’’**; *MyoRb* expression is detected in oocytes (**g**), blastomeres of cleavage-stage embryos (**g’**), and micromeres of pre- and postinversion stage embryos (**g’’**-**g’’’’**); **h–i**, *Myc* expression is detected during early oogenesis but not during embryogenesis (**h**), in adults, *Myc* is uniformly expressed in all choanocytes (**h**, **i**); **j–l** *Orphan1* expression is not detected in oocytes or embryos (**j**–**k**), but is strong in accessory cells surrounding the oocytes and embryos (**j**, **k**) and present in the uppermost (newly formed) chaonocyte chambers (**l**), *insert* in l shows a magnified young choanocyte chamber

We next studied the expression of the *Sycon* bHLH genes during development using a combination of quantitative transcriptome analysis (Fig. 2) and in situ hybridization (Fig. 3). For the transcriptome approach, we have taken advantage of the extensive RNA-seq dataset encompassing embryogenesis, metamorphosis, and adult body axis, which was previously developed for *Sycon* [26, 27, 29]. In this dataset, samples representing embryonic development were derived from mid-body slices of adult sponges containing specific oogenesis and embryogenesis stages as well as the surrounding maternal tissue. All major events of oogenesis and embryogenesis are included in this series, starting from vitellogenesis, through fertilization and cleavage, to the stages when cell differentiation (early preinversion) and morphogenesis (late preinversion and early postinversion) occur, and ending with late postinversion stage containing ready to release larvae. The metamorphosis series was derived from pools of larvae, postlarvae, and juveniles from a range of stages: i, freshly settled flat postlarvae; ii and iii, flat and spherical postlarvae with spicules; iv, formation of the first choanocyte chamber; v, opening of osculum; and ending with young syconoid sponges. Finally, expression across the adult body axis was investigated using samples derived from sections taken along the body column of non-reproductive specimens. Heatmap of expression profiles generated for the identified *Sycon* bHLH genes across all analysed samples illustrates highly regulated and very diverse temporal expression patterns (Fig. 2). To quantify the diversity of patters, we have asked whether expression of a given gene was upregulated in any of the three developmental processes: embryogenesis, metamorphosis, and adult axial patterning. The non-reproductive mid-body slices were used as the reference sample for the embryogenesis and axial patterning, while swimming larvae were used as reference for the metamorphosis comparison. All but five (*SciMlx, SciHey, SciSCL*-*like c, SciSCL*-*like b,* and *SciNSCLb*) were found to be upregulated in at least one of the developmental processes with statistical significance (padj ≤ 0.1). Expression of three genes was upregulated during all three processes, each demonstrating different expression profile: *SciOrphan1* expression peaked in late embryonic development, early metamorphosis, and the apical region of the adult sponges, *SciSCLa* in early embryonic development, early metamorphosis, and the basal part of the adult, while *SciSCLb* in the latest stages of embryonic development, late metamorphosis, and the basal part of the adult sponge (Fig. 2). Seven genes (*SciMITFa, SciSCL*-*like e, SciSCL*-*like d, SciHand, SciSCL*-*like a, SciSCL*-*like f,* and *SciOrphan3*) displayed two peaks of expression, one coinciding with embryonic development and one with metamorphosis, with the highest expression detected in various combinations of early and late steps of each of these two processes. Finally, expression of 15 genes displayed only one peak, during either embryonic development (*SciARNT*-*like, SciMyoRb, SciSREBP, SciMITFb, SciTF4, SciUSF, SciOrphan2*) or metamorphosis (*SciMyc, SciE12/E47b, SciE12/E47b, SciNSCLd, SciAtonal*-*related, SciNSCLc, SciOrphan4, SciNSCLa*), with the highest expression associated with a range of specific stages (Fig. 2). While the quantitative transcriptome analysis clearly demonstrates dynamic and diverse developmental expression of *Sycon* bHLH genes, it does not provide information regarding specific cells in which the genes are active. In addition, as the samples representing embryonic development are derived from a mixture of embryonic and maternal cells, high levels of transcripts in the adult cells (such as those seen in *SciMyc* or *SciE12/E47b*) could easily mask either upregulation or downregulation of expression in the oocytes or embryos.

We have therefore selected a subset of *Sycon* bHLH genes for a detailed expression analysis by in situ hybridization, focusing on genes representing metazoan families with conserved developmental roles: the sole representatives of Atonal, Myc, MyoR, and Hey families, four *NSCL* genes, two *SCL* genes, and one orphan gene related to the Twist family (*SciOrphan1*). We have not been able to obtain expression patterns of *SciHey*, *SciNSCLa*, and *SciNSCLb*, likely because of their relatively low expression levels as indicated by the transcriptome analysis (Fig. 2). However, analysis of the remaining nine genes revealed expression patterns consistent with conserved roles of multiple bHLH genes across the metazoans.

Almost all of the analysed genes (with exception of *SciOrphan1*, see below) are expressed in the oocytes and in all blastomeres of the cleavage-stage embryos (Fig. 3a–h, a’–g’). In pre- and postinversion stage embryos, during which cell differentiation and morphogenesis occur, expression patterns of the analysed genes become more diverse. Expression of *SciAtonal*-*like*, *SciHand*, *SciSCLb*, *SciNSCLd*, and *SciNSCLc* is particularly conspicuous in cross cells and macromeres, with *SciSCLa* transcripts detectable in macromeres only (Fig. 3a’’–f’’, a’’’–f’’’, a’’’’–f’’’’). Macromeres differentiate into the outer sponge epithelium (pinacoderm) which might be homologous with the eumetazoan ectoderm [29], reviewed by [25]. On the other hand, function of cross cells, known also as the cruciform cells or “cellules en croix” remains enigmatic. Based on dense vesicular structures observed in these cells, and the fact that they degenerate at the end of larval life coinciding with inversion of phototropism (which is positive in freshly released larvae), it has been suggested that they might be photosensory cells [36, 48], although no functional analysis has been published. *Sycon* homologs of genes involved in specification of neuronal and sensory cell types, such as transcription factors *SciSoxB*, *SciPaxB* and *SciPaxF*, *SciSixB*, and *SciHmx* as well as RNA-binding proteins Musashi and Elav, are indeed expressed in cross cells and macromeres [26–28]. Of the bHLH genes expressed in the cross cells, *Atonal, NSCL*, and *SCL* are implicated in development of sensory cells and neurons in animals including humans (e.g., [24, 49–52]), while *Hand* is involved in neural crest development (reviewed by [53]). However, as many bilaterian developmental regulatory genes, NSCL, SCL, and Hand genes perform a variety of other developmental functions, making homology assignment based on expression of a handful of genes alone difficult.

The remaining three bHLH genes are expressed in embryonic cells giving rise to the choanoderm (*SciMyoRb*, Fig. 3g’’–g’’’’), throughout the choanoderm (*SciMyc*, Fig. 3h, i) and in cells derived from choanocytes or a specific subset of choanocytes (*SciOrphan1*, Fig. 3j–l). Expression of *SciMyoRb* in micromeres is in agreement with the suggested homology of choanoderm with endomesoderm0 [29, 54], reviewed by [31, 37]. Bilaterian *MyoR* genes are expressed in muscle precursors (which are mesoderm-derived), where they antagonize function of myogenic bHLH factors such as MyoD [55]. It remains unclear what is the role of *SciMyoR*, given the absence of myogenic bHLH genes in *Sycon*, and whether this absence represents an ancestral state or is due to gene loss, a common event in the sponge lineage (reviewed by [25]).

*SciMyc* is uniformly expressed in choanocytes; its expression is elevated in young oocytes and disappears as the embryonic cells differentiate (Fig. 3h, i). In a range of bilaterian and non-bilaterian animals, *Myc* genes are implicated in stem cell specification and maintenance, cell proliferation, and gametogenesis [56, 57]. Studies in choanoflagellates (which are nearest relatives of animals) and filastereans (which are related to both choanoflagellates and animals) indicate that these roles are more ancient than the metazoans themselves [58]. *Myc*-expressing sponge choanocytes can be seen as a link between choanoflagellate-like ancestors on the one side and endodermal cells on the other; they also exhibit a variety of stem cell properties (reviewed by [31, 59]). In calcaronean sponges, choanocytes surrounding oocytes and embryos differentiate into cells assisting in fertilization and in accessory cells nourishing the embryo [33, 34]. Both of these cell types express *SciOrphan1*, which is a novel bHLH gene broadly related to the twist subfamily (Figs. 1, 3j, k). *SciOrphan1* is also expressed in choanocytes of forming radial chambers in the apical region of the sponge (Fig. 3l).

Overall, the expression patterns of bHLH genes in *Sycon* are consistent with deeply conserved roles (e.g., specification of sensory cells and stem cells by conserved genes such as *Atonal* and *Myc*, respectively) as well as novel, lineage specific functions (e.g., specification of somatic cells supporting embryogenesis by a novel family member, *SciOrphan1*).

## Conclusions

Our study demonstrates a combination of conserved and divergent features of bHLH gene families in sponges. It is increasingly clear that many independent gene loss and expansion events shaped repertoires of transcription factors in demosponge and calcisponge lineages. While some of the novel genes acquired unique expression patterns, conservation of sequence appears to be coupled with conservation of the expression specificity, implying conserved developmental roles of some bHLH across the animal kingdom.

## Additional files

10.1186/s13227-016-0060-8 Sequences of all the identified Sycon bHLH proteins.

10.1186/s13227-016-0060-8 Multiple alignment used for the phylogenetic analysis.

10.1186/s13227-016-0060-8 Matrix of gene expression levels calculated as sums of the posterior probability of each read coming from a given gene over all reads (expected_count metrics calculated with the RSEM package). First row: sample names, second row: replica names (reflecting names of the source RNASeq libraries), first column: gene identifiers.

10.1186/s13227-016-0060-8 Phylogenetic analysis of the Atonal superfamily. A rooted ML tree is shown. All the bHLH families that together constitute the Atonal-related group are indicated. Statistical supports for the nodes that define the different families and the superfamily are indicated: *** (aLRT and aBayes values > 0.95), ** (0.95 > aLRT and aBayes values > 0.8), or * (aLRT and aBayes values < 0.8).

10.1186/s13227-016-0060-8 Phylogenetic analysis of the Twist superfamily. A rooted ML tree is shown. All the bHLH families that together constitute the Twist superfamily are indicated. Statistical supports for the nodes that define the different families and the superfamily are as in Figure S1.

10.1186/s13227-016-0060-8 Phylogenetic analysis of the E12/E47 family. A rooted ML tree is shown. Statistical support for the node that defines the family is as in Figure S1.

10.1186/s13227-016-0060-8 Phylogenetic analysis of the TF4 and MLX families. A rooted ML tree is shown. Statistical supports for the nodes that define the families are as in Figure S1.

10.1186/s13227-016-0060-8 Phylogenetic analysis of the MYC, MAX, MAD, and MNT families. A rooted ML tree is shown. Statistical supports for the nodes that define the families are as in Figure S1.

10.1186/s13227-016-0060-8 Phylogenetic analysis of the SREBP, USF, and MITF families. A rooted ML tree is shown. Statistical supports for the nodes that define the families are as in Figure S1.

10.1186/s13227-016-0060-8 Phylogenetic analysis of bHLH-PAS families. A rooted ML tree is shown. Statistical supports for the nodes that define the families and some groups of families are as in Figure S1.

10.1186/s13227-016-0060-8 Phylogenetic analysis of the HEY and HES families. A rooted ML tree is shown. Statistical supports for the nodes that define the families are as in Figure S1.

## References

[1] Massari ME, Murre C (2000). Helix-loop-helix proteins: regulators of transcription in eucaryotic organisms. Mol Cell Biol.

[2] Pires N, Dolan L (2010). Origin and diversification of basic-helix-loop-helix proteins in plants. Mol Biol Evol.

[3] Sailsbery JK, Atchley WR, Dean RA (2012). Phylogenetic analysis and classification of the fungal bHLH domain. Mol Biol Evol.

[4] Simionato E (2007). Origin and diversification of the basic helix-loop-helix gene family in metazoans: insights from comparative genomics. BMC Evol Biol.

[5] Atchley WR, Fitch WM (1997). A natural classification of the basic helix-loop-helix class of transcription factors. Proc Natl Acad Sci USA.

[6] Ledent V, Vervoort M (2001). The basic helix-loop-helix protein family: comparative genomics and phylogenetic analysis. Genome Res.

[7] Sebé-Pedrós A, de Mendoza A, Lang BF, Degnan BM, Ruiz-Trillo I (2011). Unexpected repertoire of metazoan transcription factors in the unicellular holozoan *Capsaspora owczarzaki*. Mol Biol Evol.

[8] Robinson KA, Lopes JM (2000). SURVEY AND SUMMARY: *Saccharomyces cerevisiae* basic helix-loop-helix proteins regulate diverse biological processes. Nucleic Acids Res.

[9] Ponting CP, Aravind L (1997). PAS: a multifunctional domain family comes to light. Curr Biol.

[10] Fisher A, Caudy M (1998). The function of hairy-related bHLH repressor proteins in cell fate decisions. BioEssays.

[11] Suga H (2013). The Capsaspora genome reveals a complex unicellular prehistory of animals. Nat Comms..

[12] Dubois L, Vincent A (2001). The COE–Collier/Olf1/EBF–transcription factors: structural conservation and diversity of developmental functions. Mech Dev.

[13] Gyoja F (2014). A genome-wide survey of bHLH transcription factors in the Placozoan *Trichoplax adhaerens* reveals the ancient repertoire of this gene family in metazoan. Gene.

[14] Gyoja F, Satoh N (2013). Evolutionary aspects of variability in bHLH orthologous families: insights from the pearl oyster. Pinctada fucata. Zool Sci..

[15] Gyoja F, Kawashima T, Satoh N (2012). A genome wide survey of bHLH transcription factors in the coral *Acropora digitifera* identifies three novel orthologous families, pearl, amber, and peridot. Dev Genes Evol.

[16] Ledent V, Paquet O, Vervoort M (2002) Phylogenetic analysis of the human basic helix-loop-helix proteins. Genome Biol. 2002; 3(6):RESEARCH0030. 10.1186/gb-2002-3-6-research0030 PMC116727 12093377

[17] Srivastava M (2010). The *Amphimedon queenslandica* genome and the evolution of animal complexity. Nature.

[18] Degnan BM, Vervoort M, Larroux C, Richards GS (2009). Early evolution of metazoan transcription factors. Curr Opin Genet Dev.

[19] Dunn CW (2008). Broad phylogenomic sampling improves resolution of the animal tree of life. Nature.

[20] Moroz LL (2014). The ctenophore genome and the evolutionary origins of neural systems. Nature.

[21] Philippe H (2009). Phylogenomics revives traditional views on deep animal relationships. Curr Biol.

[22] Pick KS (2010). Improved phylogenomic taxon sampling noticeably affects nonbilaterian relationships. Mol Biol Evol.

[23] Pisani D (2015). Genomic data do not support comb jellies as the sister group to all other animals. Proc Natl Acad Sci U S A..

[24] Richards GS (2008). Sponge genes provide new insight into the evolutionary origin of the neurogenic circuit. Curr Biol.

[25] Fortunato SA, Adamski M, Adamska M (2015). Comparative analyses of developmental transcription factor repertoires in sponges reveal unexpected complexity of the earliest animals. Mar Genomics.

[26] Fortunato SAV, Leininger S, Adamska M (2014). Evolution of the Pax-Six-Eya-Dach network: the calcisponge case study. EvoDevo..

[27] Fortunato SAV, Adamski M, Mendivil-Ramos O, Leininger S, Liu J, Ferrier DK, Adamska M (2014). Calcisponges have a ParaHox gene and dynamic expression of dispersed NK homeobox genes. Nature.

[28] Fortunato S (2012). Genome-wide analysis of the sox family in the calcareous sponge *Sycon ciliatum*: multiple genes with unique expression patterns. EvoDevo.

[29] Leininger S (2014). Developmental gene expression provides clues to relationships between sponge and eumetazoan body plans. Nat Comms..

[30] Sebé-Pedrós A (2013). Early evolution of the T-box transcription factor family. Proc Natl Acad Sci.

[31] Adamska M (2016). Sponges as models to study emergence of complex animals. Curr Opin Genetics Dev.

[32] Amano S, Hori I (1993). Metamorphosis of calcareous sponges. 2. cell rearrangement and differentiation in metamorphosis. Invertebr Reprod Dev.

[33] Ereskovsky AV (2010). The comparative embryology of sponges.

[34] Franzen W (1988) Oogenesis and larval development of *Scypha ciliata* (Porifera, Calcarea). Zoomorphology 1988; 107:349–57.

[35] Leys SP, Eerkes-Medrano D (2005). Gastrulation in Calcareous Sponges. In Search of Haeckel’s Gastraea. Integr Comp Biol.

[36] Tuzet O (1973) Éponges calcaires. In: Grassé P-P, editor. Traité de Zoologie. Anatomie, Systématique, Biologie. Spongiaires. Masson et Cie, Paris; 1973. p. 27–132.

[37] Adamska M. Sponges as the rosetta stone of colonial-to-multicellular transition. In: KJ Niklas, SA Newman editors. Multicellularity, origins and evolution. Cambridge, MA: MIT Press. 2016. ISBN:978-0-262-03415-9.

[38] Bråte J, Adamski M, Neumann RS, Shalchian-Tabrizi K, Adamska M. Regulatory RNA at the root of animals: dynamic expression of developmental lincRNAs in the calcisponge Sycon ciliatum. Proc Biol Sci. 2015; 282(1821). 10.1098/rspb.2015.1746 PMC4707743 26702038

[39] Marchler-Bauer A, Derbyshire MK, Gonzales NR, Lu S, Chitsaz F, Geer LY, Geer RC, He J, Gwadz M, Hurwitz DI, Lanczycki CJ, Lu F, Marchler GH, Song JS, Thanki N, Wang Z, Yamashita RA, Zhang D, Zheng C, Bryant SH (2015) CDD: NCBI’s conserved domain database. Nucleic Acids Res. 2015; 43(Database issue):D222-6. doi:10.1093/nar/gku1221. 10.1093/nar/gku1221 PMC4383992 25414356

[40] Edgar RC (2004). MUSCLE: multiple sequence alignment with high accuracy and high throughput. Nucleic Acids Res.

[41] Guindon S, Gascuel O (2003). A simple, fast, and accurate algorithm to estimate large phylogenies by maximum likelihood. Syst Biol.

[42] Guindon S, Lethiec F, Duroux P, Gascuel O (2005). PhyML Online–a web server for fast maximum likelihood-based phylogenetic inference. Nucleic Acids Res.

[43] Le SQ, Gascuel O (2008). An improved general amino acid replacement matrix. Mol Biol Evol.

[44] Anisimova M, Gascuel O (2006). Approximate likelihood-ratio test for branches: a fast, accurate, and powerful alternative. Syst Biol.

[45] Anisimova M, Gil M, Dufayard JF, Dessimoz C, Gascuel O (2011). Survey of branch support methods demonstrates accuracy, power, and robustness of fast likelihood-based approximation schemes. Syst Biol.

[46] Li B, Dewey C (2011). RSEM: accurate transcript quantification from RNA-Seq data with or without a reference genome. BMC Bioinformatics.

[47] Anders S, Huber W (2010). Differential expression analysis for sequence count data. Genome Biol.

[48] Duboscq O, Tuzet O (1941). Sur les cellules en croix des Sycon (Sycon cilliatum Fabr., Sycon coronatum Ellis et. Sol., Sycon elegans Bower) et leur signification. Arch Zool Exp Gen.

[49] Ben-Arie N, McCall AE, Berkman S, Eichele G, Bellen HJ, Zoghbi HY (1996). Evolutionary conservation of sequence and expression of the bHLH protein Atonal suggests a conserved role in neurogenesis. Hum Mol Genet.

[50] Begley CG, Lipkowitz S, Gobel V, Mahon KA, Bertness V, Green AR, Gough NM, Kirsch IR (1992). Molecular characterization of NSCL, a gene encoding a helix-loop-helix protein expressed in the developing nervous system. Proc Natl Acad Sci USA.

[51] Kim WY (2012) NeuroD1 is an upstream regulator of NSCL1. Biochem Biophys Res Commun. 2012; 419(1):27–31. 10.1016/j.bbrc.2012.01.100 22310718

[52] van Eekelen JA, Bradley CK, Gothert JR, Robb L, Elefanty AG, Begley CG, Harvey AR (2013). Expression pattern of the stem cell leukaemia gene in the CNS of the embryonic and adult mouse. Neuroscience.

[53] Firulli AB (2003). A HANDful of questions: the molecular biology of the heart and neural crest derivatives (HAND)-subclass of basic helix-loop-helix transcription factors. Gene.

[54] Haeckel E (1870). On the organization of sponges and their relationship to the corals. Ann Mag Nat Hist.

[55] Lu J, Webb R, Richardson JA, Olson EN (1999). MyoR: a muscle-restricted basic helix-loop-helix transcription factor that antagonizes the actions of MyoD. Proc Natl Acad Sci U S A..

[56] Hartl M, Mitterstiller AM, Valovka T, Breuker K, Hobmayer B, Bister K (2010). Stem cell-specific activation of an ancestral myc protooncogene with conserved basic functions in the early metazoan Hydra. Proc Natl Acad Sci U S A..

[57] Hartl M, Glasauer S, Valovka T, Breuker K, Hobmayer B, Bister K (2014). Hydra myc2, a unique pre-bilaterian member of the myc gene family, is activated in cell proliferation and gametogenesis. Biol Open..

[58] Young SL, Diolaiti D, Conacci-Sorrell M, Ruiz-Trillo I, Eisenman RN, King N (2012). Premetazoan ancestry of the Myc-Max network. Mol Biol Evol.

[59] Funayama N (2013). The stem cell system in demosponges: suggested involvement of two types of cells: archeocytes (active stem cells) and choanocytes (food-entrapping flagellated cells). Dev Genes Evol.

