# Supplementary figures and images for: Conservation and divergence of bHLH genes in the calcisponge Sycon ciliatum
Source: EvoDevo. 2016 Oct 14;7:23. doi: 10.1186/s13227-016-0060-8 (PMC5064789; doi:10.1186/s13227-016-0060-8)

Other bHLH families

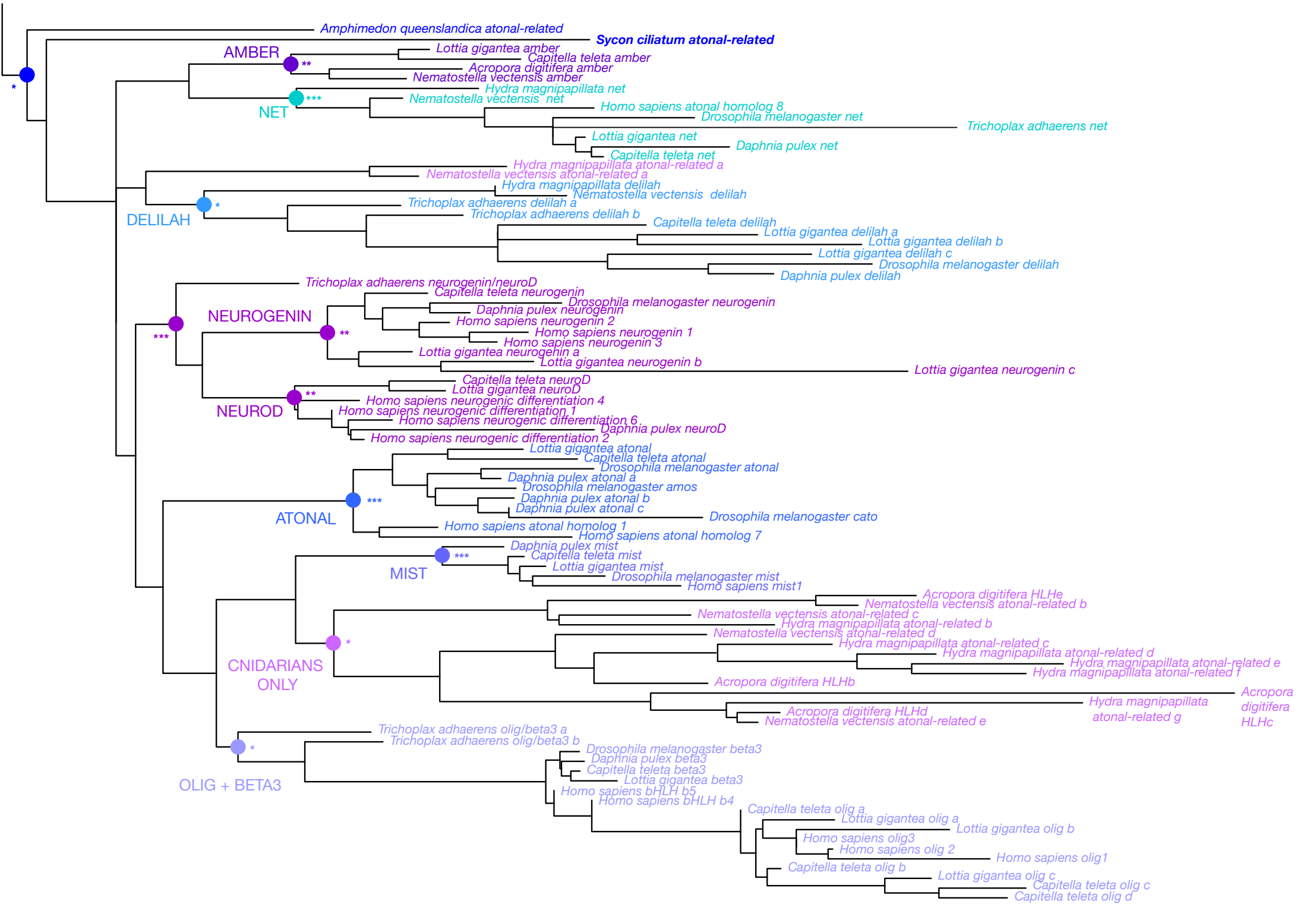

Supplement: Supplementary file 4 — 10.1186/s13227-016-0060-8 Phylogenetic analysis of the Atonal superfamily. A rooted ML tree is shown. All the bHLH families that together constitute the Atonal-related group are indicated. Statistical supports for the nodes that define the different families and the superfamily are indicated: *** (aLRT and aBayes values > 0.95), ** (0.95 > aLRT and aBayes values > 0.8), or * (aLRT and aBayes values < 0.8). [file 13227_2016_60_MOESM4_ESM.pdf]

Other bHLH families

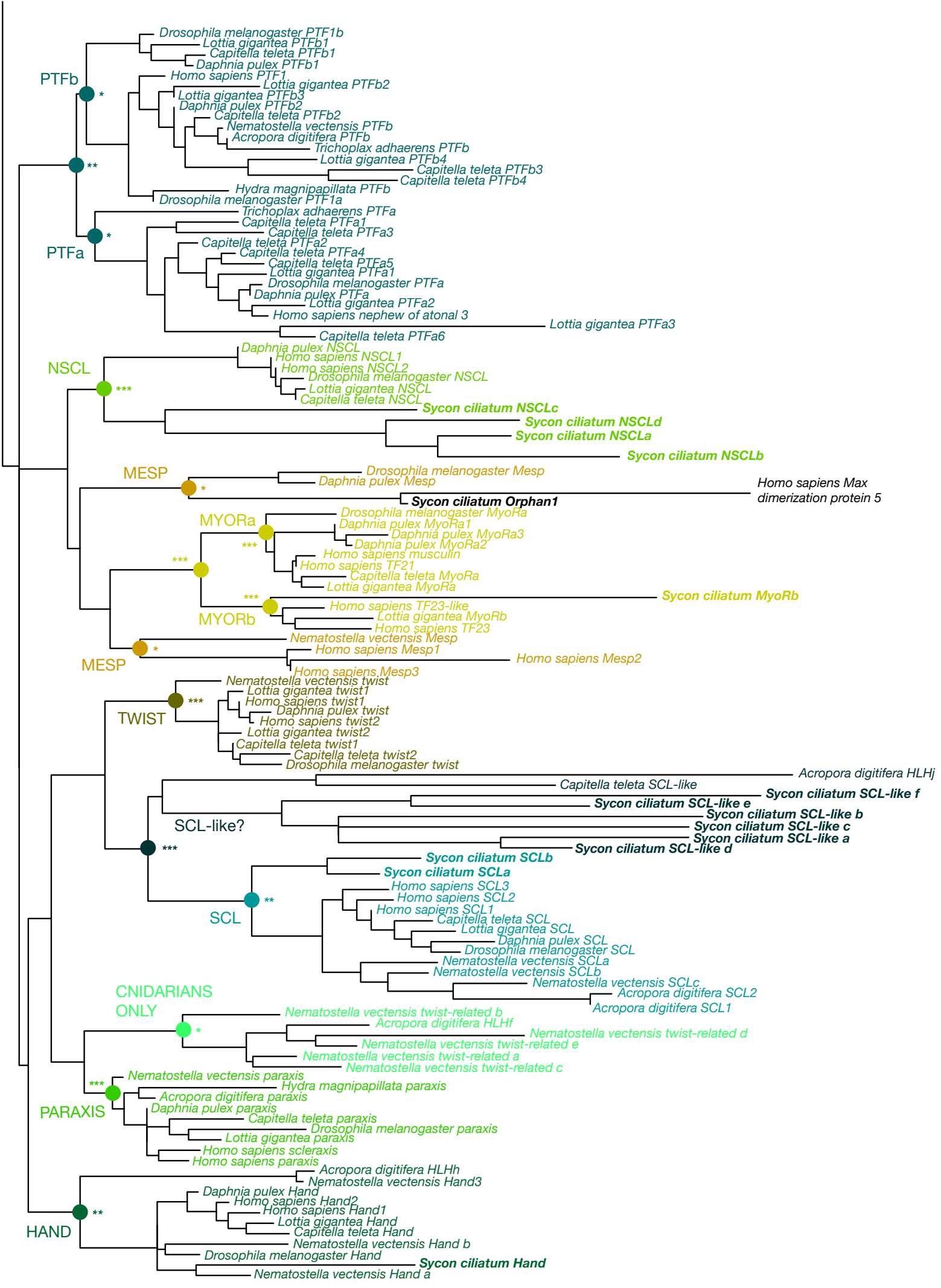

Supplement: Supplementary file 5 — 10.1186/s13227-016-0060-8 Phylogenetic analysis of the Twist superfamily. A rooted ML tree is shown. All the bHLH families that together constitute the Twist superfamily are indicated. Statistical supports for the nodes that define the different families and the superfamily are as in Figure S1. [file 13227_2016_60_MOESM5_ESM.pdf]

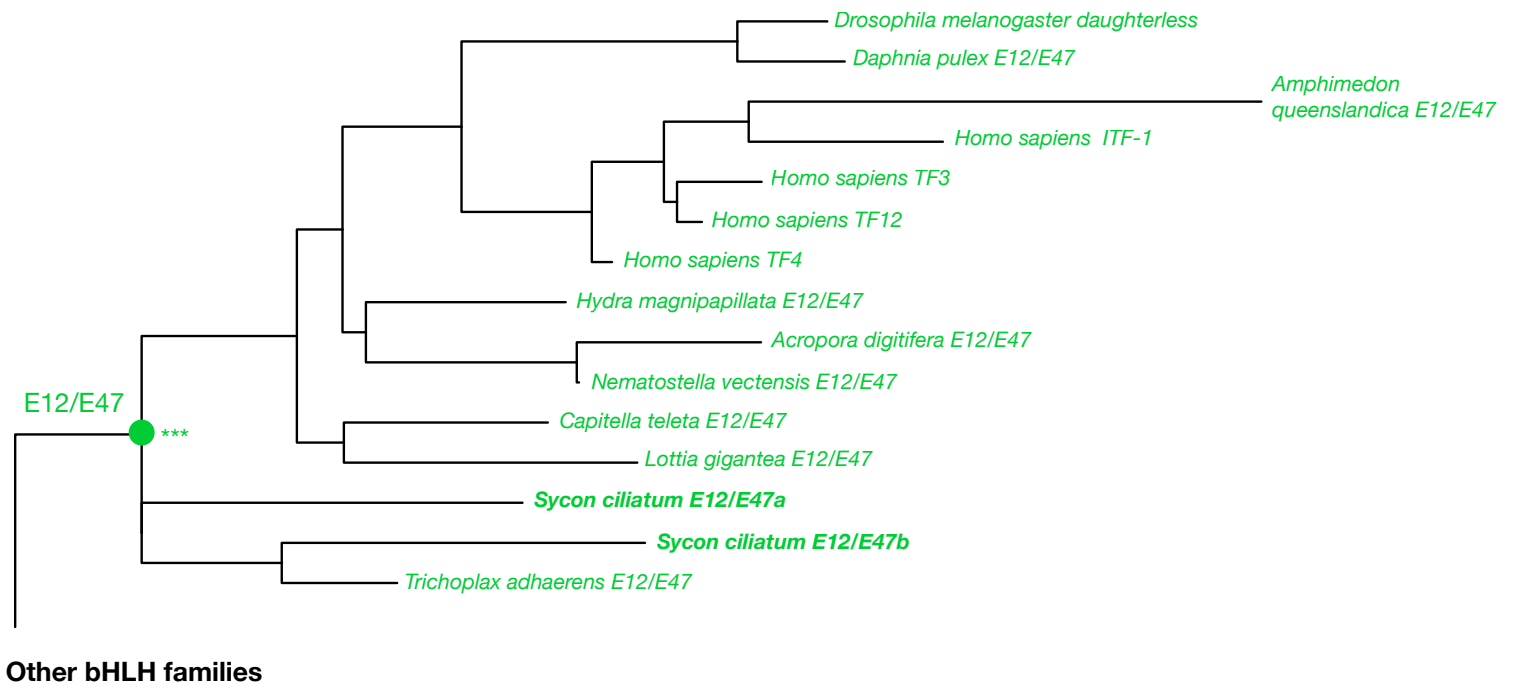

Supplement: Supplementary file 6 — 10.1186/s13227-016-0060-8 Phylogenetic analysis of the E12/E47 family. A rooted ML tree is shown. Statistical support for the node that defines the family is as in Figure S1. [file 13227_2016_60_MOESM6_ESM.pdf]

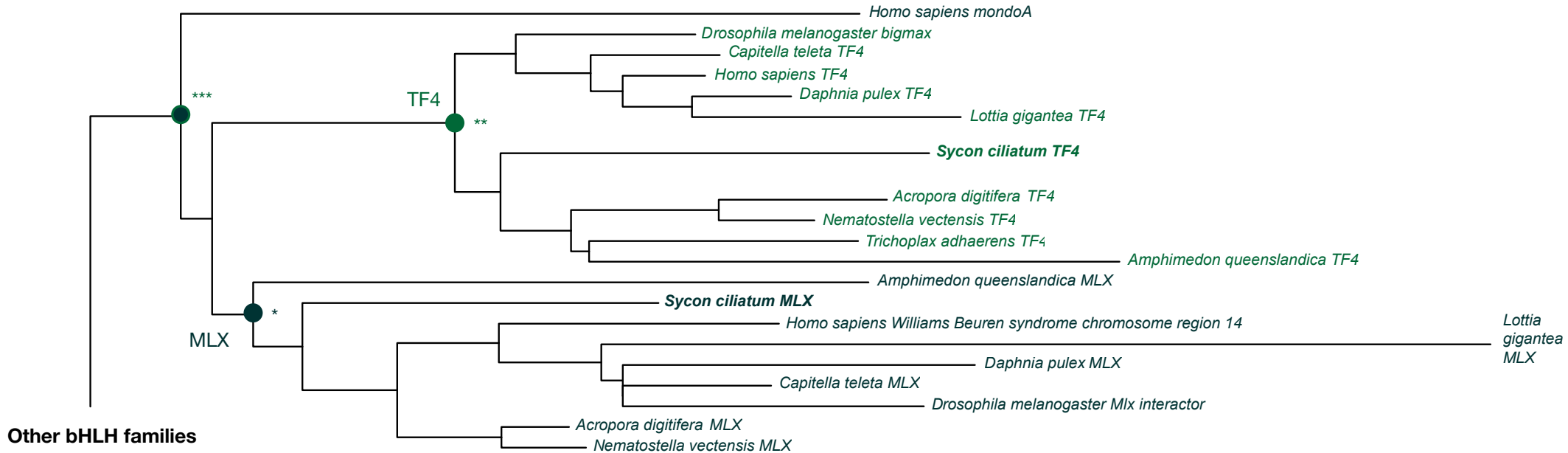

Supplement: Supplementary file 7 — 10.1186/s13227-016-0060-8 Phylogenetic analysis of the TF4 and MLX families. A rooted ML tree is shown. Statistical supports for the nodes that define the families are as in Figure S1. [file 13227_2016_60_MOESM7_ESM.pdf]

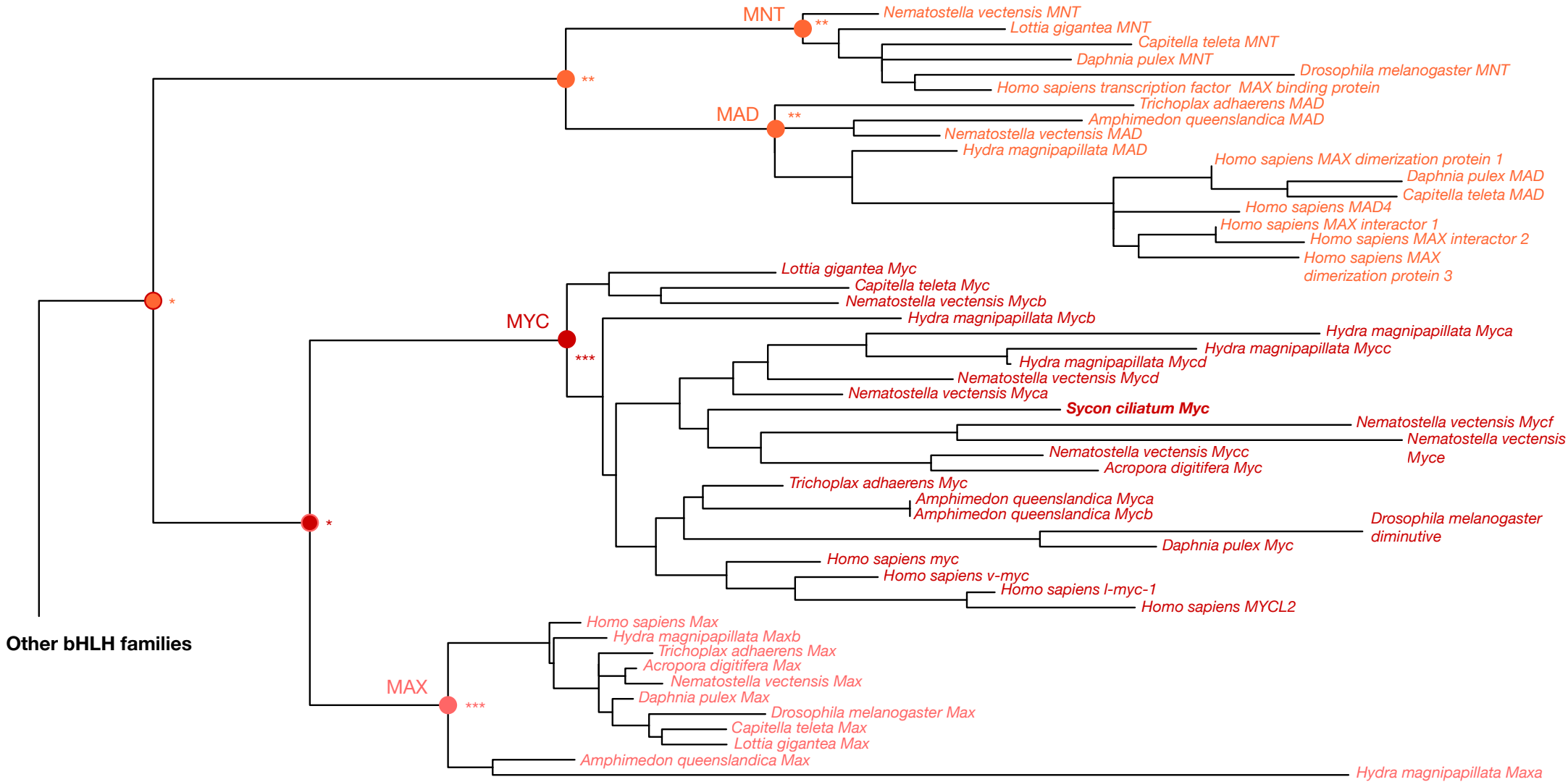

Supplement: Supplementary file 8 — 10.1186/s13227-016-0060-8 Phylogenetic analysis of the MYC, MAX, MAD, and MNT families. A rooted ML tree is shown. Statistical supports for the nodes that define the families are as in Figure S1. [file 13227_2016_60_MOESM8_ESM.pdf]

Other bHLH families

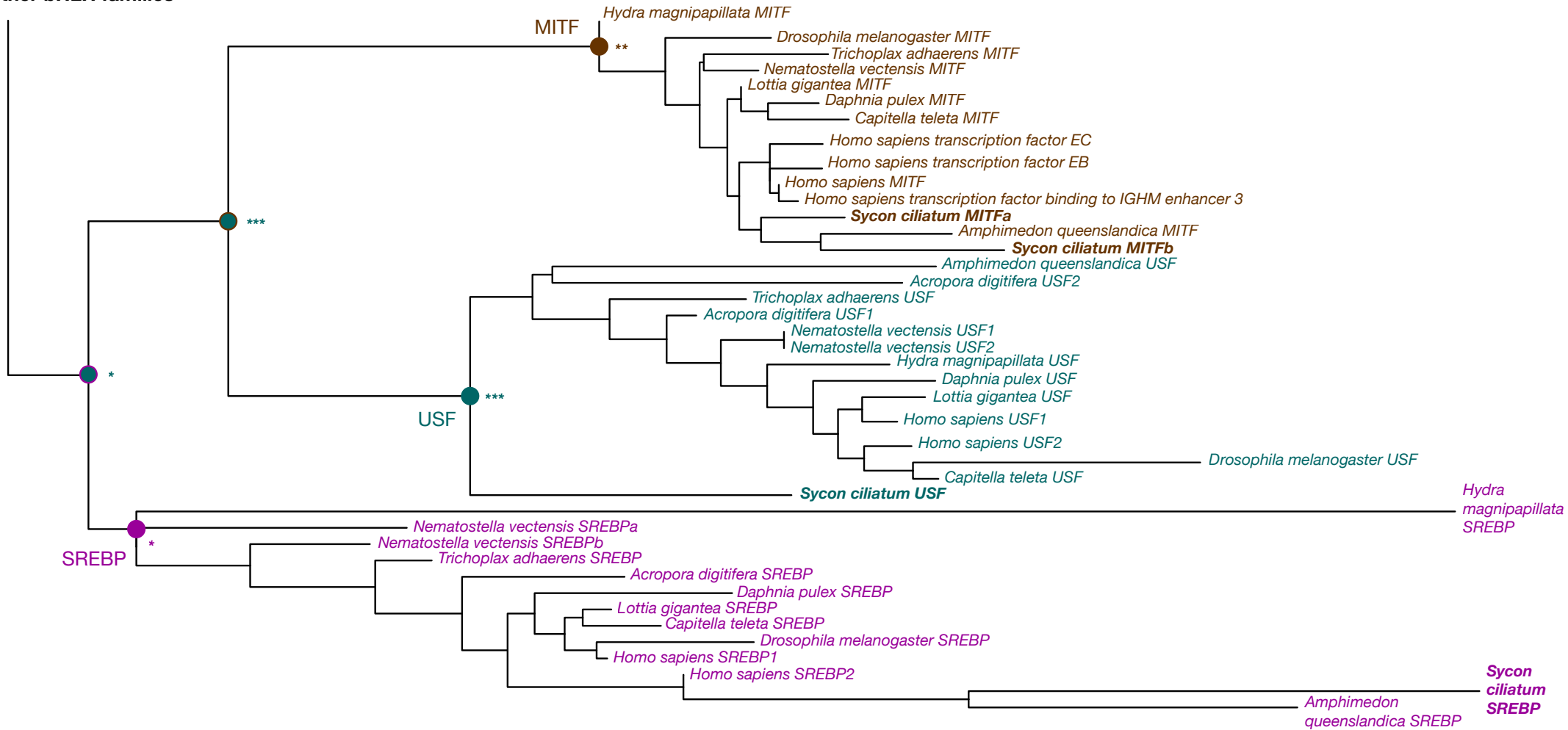

Supplement: Supplementary file 9 — 10.1186/s13227-016-0060-8 Phylogenetic analysis of the SREBP, USF, and MITF families. A rooted ML tree is shown. Statistical supports for the nodes that define the families are as in Figure S1. [file 13227_2016_60_MOESM9_ESM.pdf]

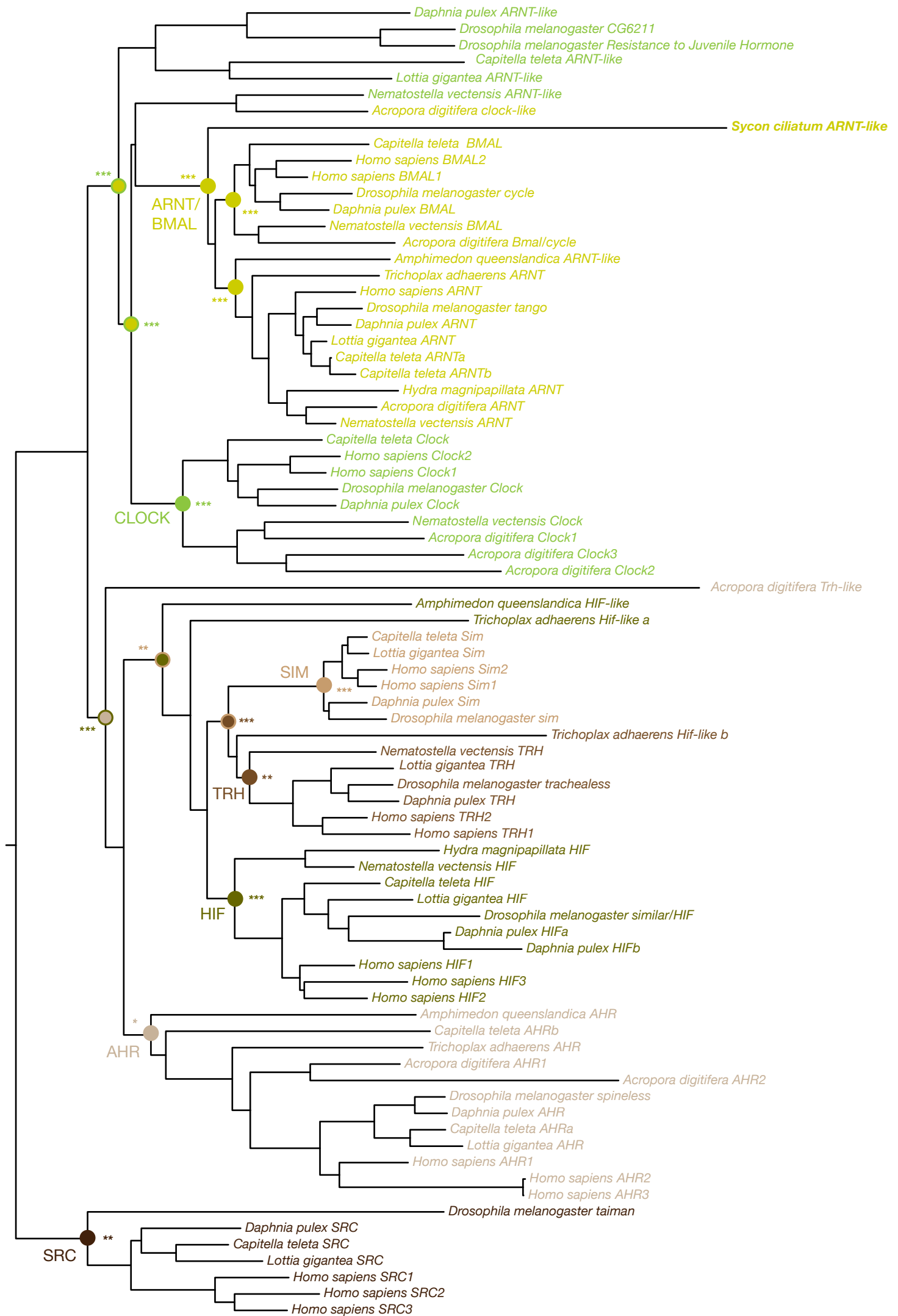

Supplement: Supplementary file 10 — 10.1186/s13227-016-0060-8 Phylogenetic analysis of bHLH-PAS families. A rooted ML tree is shown. Statistical supports for the nodes that define the families and some groups of families are as in Figure S1. [file 13227_2016_60_MOESM10_ESM.pdf]

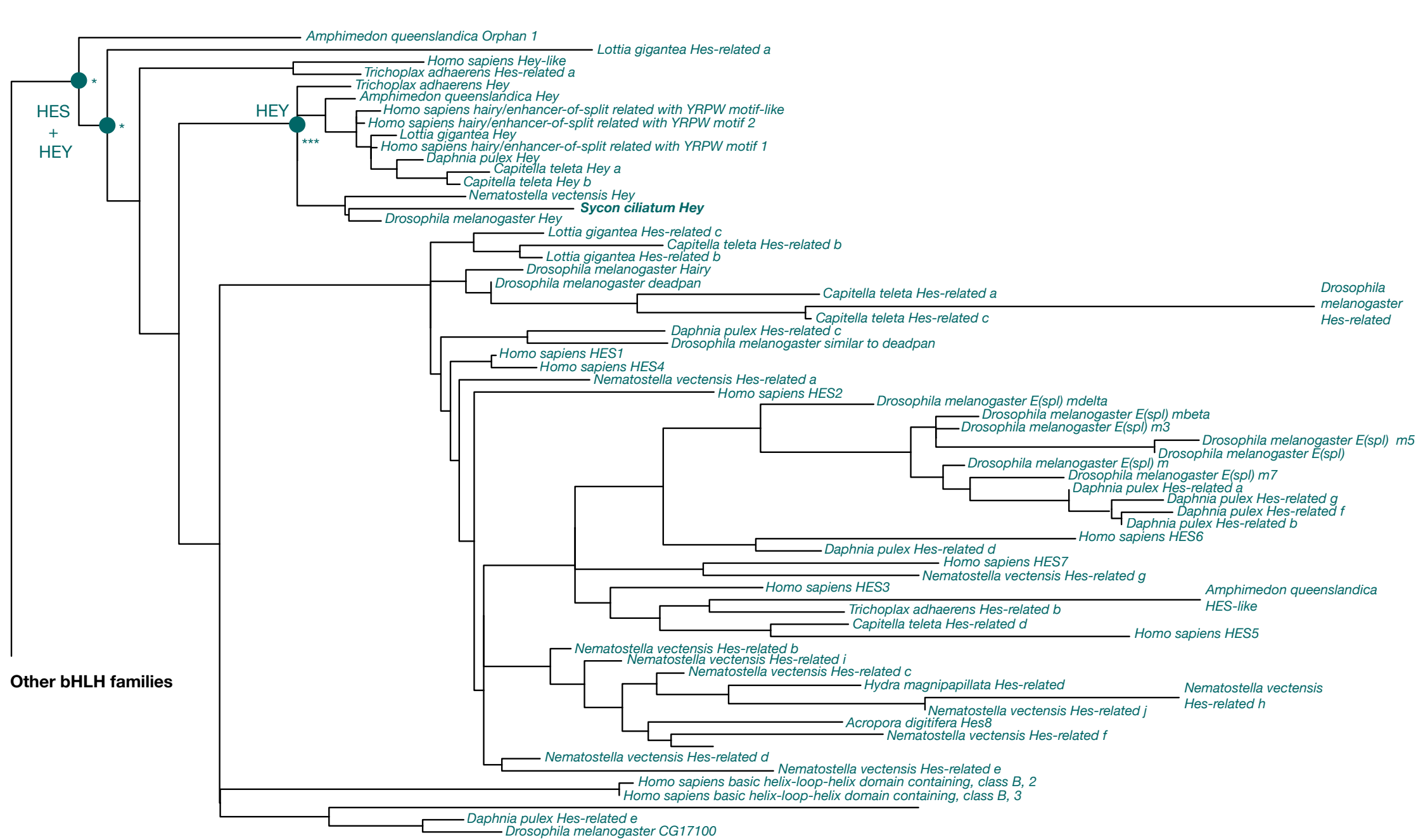

Supplement: Supplementary file 11 — 10.1186/s13227-016-0060-8 Phylogenetic analysis of the HEY and HES families. A rooted ML tree is shown. Statistical supports for the nodes that define the families are as in Figure S1. [file 13227_2016_60_MOESM11_ESM.pdf]
